# Assessment of cognitive impairment and related risk factors in hemodialysis patients

**DOI:** 10.1007/s40620-021-01170-3

**Published:** 2021-10-16

**Authors:** Hristos Karakizlis, Katharina Bohl, Jannis Ziemek, Richard Dodel, Joachim Hoyer

**Affiliations:** 1 Department of Nephrology, Philipps-University of Marburg, Marburg, Germany; 2 Department of Internal Medicine II, Division of Nephrology, Pulmonology and Critical Care Medicine, Justus-Liebig-University of Giessen, Giessen, Germany; 3 Department of Neurology, Philipps-University of Marburg, Marburg, Germany; 4 Department of Geriatric Medicine, University Hospital Essen, Essen, Germany

**Keywords:** Cognitive Impairment, CERAD, Hemodialysis, Depression, Cognitive decline

## Abstract

**Background:**

Cognitive impairment in hemodialysis patients has been acknowledged over the last years and has been reported in up to 80% of patients. Older age, high prevalence of cardiovascular risk factors, such as stroke and transient ischemic attack, uremia, and multiple metabolic disturbances represent the most common factors for cognitive impairment in hemodialysis patients.

**Methods:**

We conducted a prospective cohort study on 408 patients from 10 hemodialysis centers in the regional government district of Middle Hesse (Germany). Patients underwent a neuropsychological test battery consisting of five tests, in addition to a phonemic fluency test, to assess cognitive profile. The patients were classified as no cognitive impairment or mildly-, moderately- or severely-impaired cognitive function, depending on the degree of impairment and number of domains where the deficit was determined. We analyzed the cognitive profile and the change in performance over time in hemodialysis patients based on their cognitive status at baseline vs. 1-year follow-up.

**Results:**

Of 479 eligible patients, 408 completed all tests at baseline. Only 25% (n = 102) of the patients had no cognitive impairment. Fourteen per cent (n = 57), 36.5% (n = 149), and 24.5% (n = 100) of patients showed mild, moderate, and severe impairment, respectively. In patients with cognitive impairment, all cognitive domains were affected, and impairment was significantly associated with depression and education. The most impaired cognitive performance was immediate memory recall, and the best performance was found in naming ability. No significant  change was observed after 1-year follow up in any domain.

**Conclusion:**

Our study shows that the prevalence of cognitive impairment in hemodialysis patients is high and that it is affected by the presence of depression. Furthermore, education has an effect on cognitive test results. As depression has a significant influence on cognitive impairment, its early identification is essential in order to initiate treatment at an early stage, hoping to positively influence cognitive performance.

**Supplementary Information:**

The online version contains supplementary material available at 10.1007/s40620-021-01170-3.

## Introduction

The association of cognitive impairment with chronic kidney disease (CKD) has been reported over the last decade [1–4]. Several studies have suggested that the prevalence of cognitive impairment in patients with CKD, especially in end stage renal disease (ESRD), is up to 80% [1, 2, 5–8], but detailed data regarding different domains of cognitive functions, especially in patients with mild cognitive impairment, is scarce in literature. Early detection of cognitive impairment is of paramount significance in order to take preventive action, to assess illness-related grief, and to avoid misunderstandings during medical care [9]. Different types of dementia show different cognitive profiles. In addition to Alzheimer’s disease, where memory disorders (especially retention) dominate, vascular dementia represents one of the most common types of dementia associated with impairment in executive and parts of memory function, such as immediate recall, but not retention [10].

Hemodialysis patients are at increased risk of cognitive impairment because of their old age, high prevalence of cardiovascular risk factors, cerebrovascular involvement, including stroke and transient ischemic attack (TIA), and multiple metabolic disturbances [11–13]. Anemia has also been associated with poor cognitive function and dementia [14, 15].

Hemodialysis patients seem to suffer mostly from impairment of executive functions. Recent studies have reported associated vascular risk factors [1, 2]. Most of these studies used either the frequently applied Mini-Mental-Status-Examination (MMSE) or very heterogeneous scales. The MMSE, which is designed as a screening instrument and not as a diagnostic one, focuses on orientation, memory and language, and is, therefore, not an appropriate tool for detecting different disease-related cognitive impairments. Detailed test batteries are often time-consuming and require different standardization for each task. Therefore, a within-subject comparison in relation to performance in different cognitive domains seems to be highly required. In addition, data concerning the temporal development of cognitive impairment in hemodialysis patients are scarce, and furthermore, it remains unclear which factors might influence the development of cognitive impairment in these patients.

The goal of our study was to examine the extent of cognitive impairment, and to derive a distinct profile of cognitive function in hemodialysis patients using a standard tool for neuropsychological assessment, the Consortium to Establish a Registry for Alzheimer’s Disease (CERAD), in patients with mild cognitive impairment (MMSE Score ≥ 24). Furthermore, we explored a set of risk factors for deficits in cognitive performance as well as for its development.

## Methods

We conducted a prospective cohort study in the regional government district of Middle Hesse (Germany) at 10 outpatient dialysis centers. Inclusion criteria were: at least 18 years of age, native German speaker and receiving hemodialysis three times per week. Patients under the legal supervision of a caretaker were excluded from the study.

### Neuropsychological assessment

We applied the widely used CERAD test battery, consisting of five tests (semantic fluency, naming, verbal memory with the immediate, delayed and recognition recall subtests, constructional memory and constructional praxis) and a test of phonemic fluency, now included in the CERAD-Plus. The raw scores were transformed into z-scores using the same norm sample for all tests adjusted for age, sex and years of education.

Impairment was stratified using the algorithm adopted by Murray and colleagues [1] based on the Mayo criteria for mild cognitive impairment [16] and the Diagnostic and Statistical Manual, third Edition, Revised criteria for dementia as approximate guidelines [17]. Patients were classified depending on the amount of impairment and the number of domains the deficit was found in. Unimpaired patients performed better than 1.5 standard deviations (SD) below the norm sample in any test considered for the classification. Mildly impaired patients showed mild deficits (scores 1.50 to 1.99 SD below the norm sample) in only one domain, moderately impaired patients showed deficits in two domains or a severe deficit (2.0 SD below the norm sample) in one domain. Patients showing severe deficits in at least two domains were classified as severely impaired. The domains of executive functions (semantic and phonemic fluency), verbal memory (immediate and delayed recall), constructive praxis and language/naming were considered in the classification. As the performance in figural memory is related to the constructive skills, we did not consider the test in the classification of the patients or in further analyses. To evaluate the relationship between medical parameters and cognitive performance in general we built the CERAD-Score suggested by Chandler et al. [18]. The score combines the raw scores of the semantic fluency (limited to a maximum of 24 points), naming, immediate and delayed recall of a word list, recognition of those words and constructive praxis subtests, resulting in a total-score ranging from zero to one hundred points.

### Dialysis measures, comorbidity, laboratory parameters

Data concerning hemoglobin, creatinine, calcium, phosphorus, albumin, triglycerides, cholesterol and urea, as well as pH-value, CO_2_, bicarbonate, blood pressure, and dialysis dose were obtained. Demographic, comorbidity and current medication data were obtained from the medical records or were self-reported. The examinations, which took place within the first 90 min of dialysis therapy, were carried out by thoroughly instructed medical students.

### Statistical methods

Demographic and medical parameters were compared among hemodialysis patients with no impairment, an impairment in any domain, or a severe impairment, using *t*-tests for normally distributed variables, Mann–Whitney-U-Tests for not normally distributed variables and Fisher’s exact- or Cramer’s-V Tests for categorical variables. We analyzed the correlation between different demographic, clinical, and laboratory parameters, including dialysis duration, dialysis efficiency (estimated by the Kt/V equation), and calcium-phosphate-product, and performance in different cognitive tests (Table S1). Parameters which showed statistically significant correlations with performance at different cognitive tests (p < 0.05) were entered as possible predictors in a multivariate regression model with a backward elimination method to test for an independent association. Predictors with *p-value* < 0.10 were considered significant and were retained in the model, while others were excluded. We performed the analysis of variance (ANOVA) test with repeated-measures to analyze the cognitive profile and the change in performance over time in hemodialysis patients based on the cognitive status at baseline. The inner-subject factors *time* (baseline vs. 1-year-Follow-up) and *test* (semantic fluency, phonemic fluency, naming, memory immediate recall, memory delayed recall, memory recognition, constructive praxis), as well as the between-subject factor *cognitive impairment* (none, mild, moderate, severe at baseline) resulted in a 2 × 7 × 4 design with pairwise comparisons and planed contrasts for a priori analysis, choosing z-scores in immediate recall as reference. To explore the effect of depression on the decrease in cognitive performance, we calculated a 2 × 2 design (*time* × *depression*). The same analyses were repeated with the last observation carried forward method. All assumptions regarding multivariate regression analyses or variance analyses with repeated measures, including homoscedasticity, linearity, autocorrelation, normally distributed errors or dependent variable, multicollinearity and sphericity were accounted for by means of the appropriate methods. The Greenhouse-Geißer correction was used when the assumption of sphericity was violated. Differences were considered statistically significant at *p* values < 0.05. All analyses were performed using the Predictive Analysis SoftWare (PASW®) version 17.0 (SPSS Inc., Chicago, IL, USA) (Table 1).

**Table 1:** Characteristics and group differences between patients with and without cognitive impairment

Variable	No cognitive impairment	Cognitive impairment in any domain	Sig^1^	Severe cognitive impairment	Sig^2^
Age, mean ± sd (N)	71.6 ± 10.2 (102)	71.7 ± 9.8 (306)	.920	69.8 ± 9.0 (100)	.198
Female, % (N)	32.4% (33)	42.8% (131)	.063	44.0% (44)	.111
Years of education, mean ± SD (N)	11.5 ± 2.4 (102)	10.9 ± 2.3 (305)	.029*	11.1 ± 2.3 (100)	.197
Time on dialysis, Months, mean ± SD (N)	36.97 ± 38.66 (73)	48.63 ± 56.37 (266)	.174	49.41 ± 47.63 (70)	.100
Hours on dialysis per session, mean ± SD (N)	4.2 ± 0.8 (102)	4.3 ± 0.5 (305)	.497	4.3 ± 0.5 (100)	.519
Primary Cause of ESRD, % (N)			.879		.734
Diabetes	26.5% (27)	23.9% (73)		21.0% (21)	
Vascular	11.8 (12)	12.7% (39)		11.0 (11)	
Others	60.8 (62)	61.1% (187)		63.0% (63)	
Systolic Blood pressure, mean ± SD (N)	129.4 ± 23.2 (89)	133.1 ± 20.0 (259)	.156	134.6 ± 21.1 (83)	.137
Diastolic Blood pressure, mean ± SD (N)	65.7 ± 12.0 (89)	67.0 ± 11.9 (258)	.387	68.0 ± 12.3 (82)	.254
Pulse pressure, mean ± SD	63.7 ± 18.3 (89)	66.1 ± 17.7 (258)	.279	66.7 ± 17.1 (82)	.281
Equilibrated Kt/v, mean ± SD (N)	1.5 ± 0.4 (87)	1.6 ± 0.5 (255)	.279	1.6 ± .5 (89)	.291
Hemoglobin, mean. ± SD (N)	11.6 ± 1.3 (102)	11.6 ± 1.2 (294)	.951	11.8 ± 1.2 (94)	.316
Albumin, mean ± SD (N)	36.6 ± 5.5 (88)	36.1 ± 5.1 (263)	.395	36.3 ± 4.7 (84)	.500
Calcium phosphate product, mean ± SD (N)	3.5 ± 1.1 (101)	3.8 ± 1.3 (296)	.183	3.9 ± 1.2 (94)	.049*
Bicarbonate, mean ± SD (N)	22.3 ± 3.6 (84)	22.6 ± 3.2 (251)	.630	22.2 ± 2.9 (71)	.892
Arterial hypertension, % (N)	75.5% (77)	79.7% (244)	.188	81.0% (81)	.100
Cholesterol, mean ± SD (N)	173.5 ± 43.1 (80)	180.0 ± 98.6 (220)	.802	180.3 ± 45.8 (70)	.343
Coronary heart disease, % (N)	47.1% (48)	34.3% (105)	.044*	32.0% (32)	.060
Diabetes mellitus, % (N)	37.3% (38)	39.2% (120)	.639	39.0% (39)	.660
Stroke or TIA, % (N)	10.8% (11)	11.8% (36)	.859	13.0% (13)	.664
Atrial fibrilation, % (N)	19.6% (20)	18.6% (57)	1.0	13.0% (13)	.340
Nicotine use, % (N)	38.2% (39)	31.7% (97)	.275	27.0% (27)	.102
Alcohol use, % (N)	5.9% (6)	4.9% (15)	.575	6.0% (6)	.501
MMSE. mean ± SD (N)	28.2 ± 1.6 (102)	27.4 ± 1.8 (306)	.001***	26.9 ± 1.9 (100)	.001***
None CI (28–30) % (N)	72.5% (74)	50.3% (154)		44.0% (44)	
Mild CI (25–27) % (N)	25.5% (26)	42.2% (129)		41.0% (41)	
Moderate CI (20–24) % (N)	2.0% (2)	7.5% (23)		15.0% (15)	
CERAD Score, % (N)	77.7 ± 7.9 (102)	64.4 ± 10.0 (306)	.001***	59.6 ± 9.5 (79)	.001***
Dementia, % (N)	0% (0)	0.7% (2)	.550	0.0% (0)	
Depression, % (N)	3.9% (4)	9.8% (30)	.064	10.0% (10)	.096

Bold print significance level *p < .05; **p < .01; ***p < .001

SD = standard deviation; ^1^Comparison between patients with non cognitive impairment and those with mild cognitive impairment, ^2^ Comparison between patients with no cognitive impairment and those with severe cognitive impairment

## Results

### Patient characteristics

Out of 629 screened patients, 153 did not fulfill the inclusion criteria. Seventy eligible patients either refused (n = 67) or were not able to complete the neuropsychological testing (n = 3). Those patients had significantly fewer years of education, lower scores in the MMSE, higher diastolic blood pressure, higher values of hemoglobin and bicarbonate, relatively more coronary heart diseases, and lower smoking index. One hundred sixty-five patients were lost to follow-up. There were no significant differences in demographic or medical parameters at baseline between the excluded or attrited patients and those who completed the study, except for a higher prevalence of atrial fibrillation in excluded or attritted patients. No difference was found at baseline regarding test performance between the patients who completed the study and those who were lost to follow up.

Patients in the study group (n = 408) were on average 71.6 years (SD: 10.29) old at baseline and had a mean of 11.1 years (SD: 2.39) of education (school and professional education in total). Forty point two% of them were women, and the mean time on dialysis was 48.4 months (SD: 77.04). Thirty nine point eight% had diabetes; most of the patients were suffering from arterial hypertension (78.7%), 11.5% had a history of stroke or TIA and 8.3% had a diagnosis of depression.

### Cognitive impairment

Only 25% (n = 102) of the patients were cognitively unimpaired. Fourteen percent (n = 57) had mild, 36.5% (n = 149) moderate and 24.5% (n = 100) severe impairment. Table 2 shows the frequency of cognitive impairment in the different tasks. The most prevalent impairment appeared in immediate memory recall as well as in phonemic and semantic fluency.

**Table 2:** The rate of cognitive impairment at baseline and at one year follow-up

	Domains	Subtests	Raw scores	Impairment		
				None	Mild*	Severe**
Baseline	Executive functions	Semantic fluency	**15.27 ± 5.19**	**69.3%**	**15.7%**	**15.0%**
		Phonemic fluency	**7.27 ± 4.14**	**67.2%**	**12.5%**	**20.3%**
	Language	Naming	**13.42 ± 1.67**	**85.0%**	**6.4%**	**8.6%**
	Verbal memory	Immediate memory recall	**16.27 ± 4.34**	**59.1%**	**15.9%**	**25.0%**
		Delayed memory recall	**5.16 ± 2.37**	**76.4%**	**10.0%**	**13.5%**
		Memory recognition	8.46 ± 1.77	70.0%	11.8%	18.1%
	Constructive praxis	Constructive praxis	**9.35 ± 1.53**	**71.1%**	**10.3%**	**18.6%**
1 year-Follow-up	Executive functions	Semantic fluency	**15.26 ± 5.43**	**69.5%**	**17.7%**	**12.8%**
		Phonemic fluency	**7.37 ± 4.06**	**65.8%**	**13.2%**	**21.0%**
	Language	Naming	**13.42 ± 1.72**	**85.2%**	**8.2%**	**6.6%**
	Verbal memory	Immediate memory recall	**16.30 ± 4.74**	**64.2%**	**11.5%**	**24.3%**
		Delayed memory recall	**5.34 ± 2.35**	**76.5%**	**12.3%**	**11.1%**
		Memory recognition	8.45 **± 1.97**	74.1%	10.7%	15.2%
	Constructive praxis	Constructive praxis	**9.34 ± 1.45**	**72.0%**	**11.5%**	**16.5%**

Comparison between the frequency of cognitive impairment in patients at baseline (top row) and after one year of follow-up (bottom row). In analysis of variance (ANOVA) with repeated-measures, no significant difference in z-scores between baseline cognitive profile and those at 1-year follow up in any domain was found, suggesting no significant main effect of *time* (F(1, 239) = 2.264; *p* = 0.134)

^*^-1.99 ≤ z-score ≤ -1.5; ** z-score ≤ -2.0. **Bold print:** considered in the classification

### Non-dialysis specific factors and their association with cognitive test performance

In Table 1 the patients’ characteristics are listed separately for the groups of; no cognitive impairment, at least mild deficits in any considered tests, and severe cognitive impairment. Patients with no cognitive impairment had significantly more years of education and higher prevalence of coronary heart disease compared to those with mild cognitive impairment. Furthermore, female gender and depression tended to be more frequent in the group with mild cognitive impairment compared to the cognitively sound group (p = 0.063, 0.064, respectively). Compared to patients with severe cognitive impairment, cognitively unimpaired patients showed a significantly lower calcium phosphate product (0.049). Interestingly, dialysis duration (months) did not differ among the three groups (*p* = NS).

Multivariate regression analysis was conducted to investigate an independent association between age, gender, years of education, hemoglobin level, smoking (pack-year), stroke/TIA, depression, or hypertension and performance in different aspects of cognitive tests (Table 3).

**Table 3:** Multivariate multiple regression analysis showing factors affecting performance in different cognitive tests

	semantic fluency	phonemic fluency	immediate memory recall	delayed memory recall	memory recognition	Naming	constructive praxis	CERAD-Score
	β (95% CI)	β (95% CI)	β (95% CI)	β (95% CI) /	β (95% CI)	β (95% CI)	β (95% CI)	β (95% CI)
Age (years)	– 0.07** (– 0.12– – 0.02)		– 0.148*** (– 0.189– – .107)	– 0.080*** (– 0.102– – 0.057)	– 0.051*** (– 0.068, – 0.035)	0.03*** (– 0.046– – 0.014)	– 0.014 (– 0.029– 0.001)	– 0.395*** (– 0.494– – 0.296)
Years of education	0.541*** (0.327– 0.756)	0.428*** (0.259– 0.597)	0.196* (0.019– 0.373)	0.115* (0.026– 0.014)	0.152*** (0.083–0.220)	0.142*** (0.076, 0.207)	1.107*** (0.678– 1.535)	0.152*** (0.083– 0.220)
Sex (male vs. female)				0.821*** (0.366– 1.277)			– 0.620*** (– 0.929, – 0.310)	
Hemoglobin (g/dl)	0.627** (0.241– 1.041)							
Depression(vs. no depression)				– 1.062** (– 1.825– – 0.299)		– 0.541 (.– 1.107– 0.025)		– 4.313* (– 7.786, – 0.839)
Arterial hypertension (vs. Normotension)						– 0.403 (– .806, 0)		
Smoking (pack– year)		1.146** (0.316– 1.976)					0.264 (– 0.050–0.577)	
TIA / Stroke								– 2.902 (– 5.938– 0.135)

β: unstandardized regression coefficient; 95% CI: confidence interval;; *p < .05; **p < .01; ***p < .001

Age showed, as expected, an independent negative association with semantic fluency, early and late memory recall, naming ability, constructive praxis, and CERAD score. Unlike age, years of education showed an independent positive association with all aforementioned cognitive aspects in addition to phonemic fluency (Table 3). Males showed better delayed memory recall but worse constructive praxis than females (β = 0.821, *p* < 0.001; β = – 0.62, *p* < 0.001, respectively). Hemoglobin level was independently positively associated with semantic fluency (β = 0.627, *p* < 0.01), but showed no significant correlation with other cognitive domains (*p* = NS). Depression showed an independent negative association with delayed memory recall and CERAD score (β = – 1.062, *p* < 0.01; β = – 4.3. *p* < 0.05, respectively). Although hypertension showed a significant correlation with delayed memory recall and naming ability, the association did not remain significant after adjusting for confounding factors (*p* = NS) (Tables S1 and 3). Interestingly, smoking showed an independent positive association with phonemic fluency (β = 1.146, p < 0.01). History of stroke or TIA did not show a significant association with performance in cognitive tests (*p* = NS).

### Dialysis-specific factors and their association with performance in cognitive tests

**Fig. 1:**
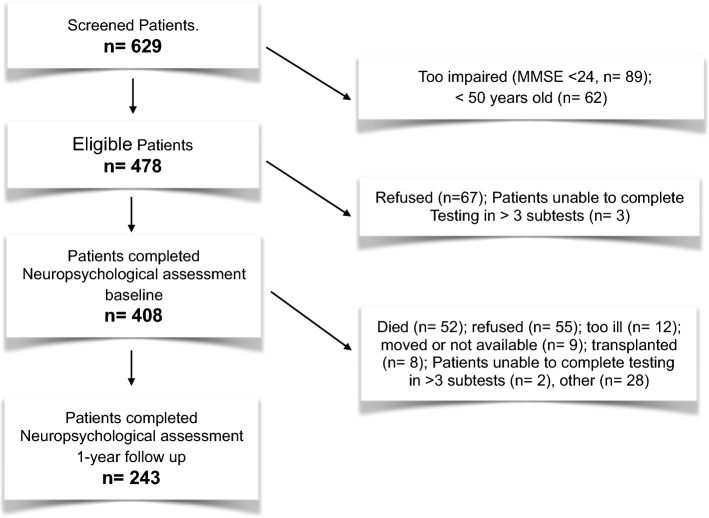
Flow chart of the study

**Fig. 2:**
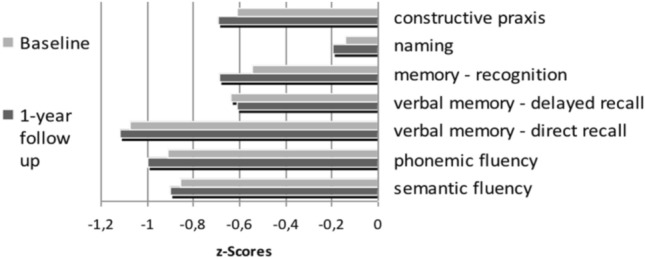
Cognitive profile of hemodialysis patients. The cognitive profile at baseline and at 1-year-follow up for all patients is shown. In analysis of variance (ANOVA) with repeated-measures, we found no significant difference in z-scores between baseline cognitive profile and those at 1-year follow up in any domain, suggesting no significant main effect of *time* (F(1, 239) = 2.264; *p* = 0.134)

Neither dialysis duration (months) nor duration of dialysis session (hours) showed a significant association with performance in the aforementioned cognitive tests (p = NS) (Table S1). Likewise, dialysis efficiency, estimated with equilibrated Kt/v, and calcium-phosphate-product showed no association with performance in cognitive tests. As shown in Table 1, duration of dialysis (months), duration of dialysis session (hours), and equilibrated Kt/v did not differ significantly among the 3 study groups. Calcium-phosphate-product was marginally significantly higher in the group with severe cognitive impairment compared with that with no cognitive impairment (*p* = 0.049) (Figs. 1, 2).

### Cognitive profile of hemodialysis patients and its development over one year

**Fig. 3:**
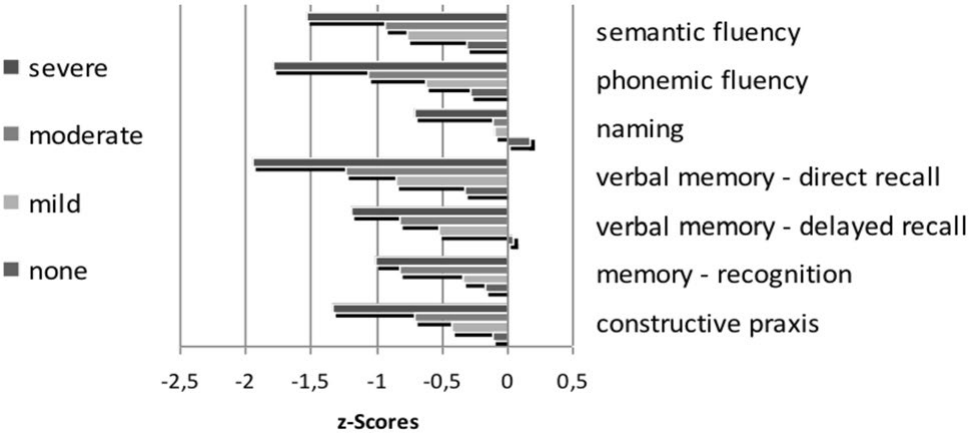
*Group-by-time* interaction effect (F(3, 239) = 3.196, *p* < 0.05), as shown by a different development of z-scores at one-year follow-up in relation to the grade of cognitive impairment at baseline (differences in z-values between baseline and 1-year follow-up). All domains reveal an impairment in cognitive performance. Bonferroni-corrected pairwise comparisons indicate that patients had the lowest scores in immediate recall (dr), phonemic (pf) and semantic fluency (sf),while no significant effects were found between those three (dr vs. pf: *p* = 1.0; dr vs. sf: *p* = .289; pf vs. sf: *p* = 1.0). All z-scores in the three tests were significantly lower compared to z-scores in the subtests of delayed recall (der; vs. dr: *p* < .001; vs. pf: *p* < .01; vs. sf: *p* < .05), memory recognition (mr; vs dr: *p* < .001; vs. pf: *p* < .01; vs. sf: *p* < .05), naming (na; vs dr: *p* < .001; vs. pf: *p* < .001; vs. sf: *p* < .001) and constructive praxis (cp; vs. dr: *p* < .001; vs. pf: *p* < .05). Only the difference between the semantic fluency and constructive praxis subtests failed significance. der: delayed recall; dr: direct recall, pf: phonemic fluency, sf: semantic fluency, mr: memory recognition, cp: constructive praxis, naming: na

ANOVA with repeated-measures showed no significant difference in z-scores between baseline cognitive profile and those at 1-year follow up, suggesting no significant short-term effect of *time* (F(1, 239) = 2.264; *p* = 0.134) However, we found a significant *group-by-time* interaction effect (F(3, 239) = 3.196, *p* < 0.05), as shown by a different development of z-scores at one-year follow-up in relation to the grade of cognitive impairment at baseline (differences in z-values between baseline and 1-year follow-up: no cognitive impairment group: – 0.192; mild cognitive impairment = – 0.027; moderate cognitive impairment = – 0.062; severe cognitive impairment = 0.077). The main effect *test* presents significant differences in performance among subtests (F(4.60, 1099.63) = 24.439; *p* < 0.001). Bonferroni-corrected pairwise comparisons indicated that patients had the lowest scores in immediate recall (dr), phonemic (pf) and semantic fluency (sf). While no significant effects were found between these three items (dr vs. pf: *p* = 1.0; dr vs. sf: *p* = 0.289; pf vs. sf: *p* = 1.0), all z-scores in the three tests were significantly lower compared to z-scores in the following subtests; delayed recall (der; vs. dr: *p* < 0.001; vs. pf: *p* < 0.01; vs. sf: *p* < 0.05), memory recognition (mr; vs dr: *p* < 0.001; vs. pf: *p* < 0.01; vs. sf: *p* < 0.05), naming (na; vs dr: *p* < 0.001; vs. pf: *p* < 0.001; vs. sf: *p* < 0.001), constructive praxis (cp; vs. dr: *p* < 0.001; vs. pf: *p* < 0.05). Only the difference between the semantic fluency and constructive praxis subtests failed significance. The results are illustrated in Fig. 3.

The highest scores were reached in the naming test (vs. der: *p* < 0.001; vs. mr: *p* < 0.01; vs. cp: *p* < 0.001). There was significant interaction between the factor *test* and *cognitive impairment* at baseline, F (13.80, 1099.63) = 1.726, *p* < 0.05, indicating that the profile was mostly shaped through more severely impaired patients. We also found a significant main effect for the amount of cognitive impairment at baseline, F(3, 239) = 71.181, *p* < 0.001. Contrasts were performed comparing each test to z-scores in the immediate memory recall subtest across the different impairment groups. These revealed a significant interaction when comparing the different cognitively impaired groups at baseline. The following interactions were found: Immediate memory recall compared with tests of (1) delayed memory recall (F[3, 239] = 3.551; *p* < 0.05), (2) memory recognition (F[3, 239] = 4. 788; *p* < 0.01), and (3) on naming (F[3, 239] = 3.55; *p* < 0.05), but no interactions were found on (4) semantic fluency (F[3, 239] = 1.523; *p* = 0.209), (5) phonemic fluency (F[3, 239] = 0.299; *p* = 0.876), or (6) constructive practice (F[3, 239] = 0.942; *p* = 0.421). Therefore, the results in the pairwise comparison show that the patients who were already severely cognitively impaired at baseline continued to decline in cognitive performance in the follow-up examination.

To recapitulate the previous results, the amount of decrease in cognitive performance differs depending on the starting level. The cognitive profile showing the worst results in immediate memory recall, phonemic and semantic fluency and the best in naming, is more distinctive in patients that were more severely impaired.

Therefore, we performed further ANOVAs with repeated-measures separately for the different degree of impairment at baseline. As presented in table S2, there was only a significant main effect of *time* in patients who were not impaired at 1-year follow-up (F[1,68] = 13.311, *p* < 0.01), but not in the other groups (mild impairment: F[1, 31] = 0.096, *p* = 0.758; moderate impairment: F[1,83] = 1.341, *p* = 0.250; severe impairment: F[1,57] = 1.100, *p* = 0.299). The known main effect of the factor *tests* was found in all groups (no impairment: F[4.825,328.076] = 6.182, *p* < 0.01; mild impairment: F[4.685, 145.245] = 4.056, *p* < 0.01; moderate impairment: F[3.879, 321.923] = 11.381, *p* < 0.001; severe impairment: F[4.585, 261.368] = 11.538, *p* < 0.001). The interaction between the factors *time* and *test* failed significance in all groups (no impairment: F[5.074, 345.045] = 1.276, *p* = 0.273; mild impairment: F[4.469, 138.544] = 1.788, *p* = 0.127; moderate impairment: F[5.329, 442.267] = 0.479, *p* = 0.803; severe impairment: F[5.159, 294.05] = 0.815, *p* = 0.543).

To explore the differences in the decrease of cognitive impairment in a more detailed manner, we performed paired-sampled *t*-tests separately for the cognitive status at the beginning of this study. We used the CERAD-Score as an independent variable because we had already explored the cognitive profile and there was no sign of different change over time depending on the test. The differences between the CERAD-scores at baseline compared to the scores one year later were only significant in patients with no impairment at baseline (*t*[64] = 3.170, *p* < 0.01, mean difference [md] = 2.74 [SD: 6.94; CI: 1.01, 4.46]), but not in the other groups (mild impairment: *t*[30] = – 0.984, *p* = 0.333, md = – 1.06 [SD: 6.02; CI: – 3.27, 1.14]; moderate impairment: *t*[82] = 0.806, *p* = 0.423, md = 0.73 [SD: 8.31; CI: – 1.08, 2.55]; severe impairment: *t*[57] = 0.000, *p* = 1.0, md = 0 [SD: 7.40; CI: – 1.95, 1.95].

Analyses using the last observation carried forward method confirmed the results, except for significant differences in the performance in semantic fluency and constructive praxis, and slight differences in planed contrasts.

Depression was the most influential variable next to age and education in the regression analyses. Therefore, we analyzed whether depression also had an influence on the development in cognitive performance over 1 year, again using the CERAD-Score as an independent variable. We calculated the 2 × 2 ANOVA (time × depression) with repeated-measures for the whole sample because of the small number of depression diagnoses.

There was a significant main effect for *time* (F[1, 235] = 16.539, *p* < 0.001), but even more interestingly, there was also a significant interaction between *time* and *depression*, F(1, 235) = 13.363, *p* < 0.001). This indicates a decrease in cognitive performance for people with depression (Mean = 62.89, SD = 10.27; Mean = 55.94, SD = 11.63), but not for patients without (Mean = 69.20, SD = 10.77; Mean = 68.83, SD = 12.14).

We also found the expected main effect of *depression*, F(1, 235) = 13.061, *p* < 0.001, showing lower CERAD-Scores at baseline and at 1-year follow-up in patients with diagnosed depression compared to those without. Analysis with last observation carried forward did not reveal different results.

## Discussion

We used a comprehensive neuropsychological test battery (CERAD) to examine the cognitive profile of cognitive function in hemodialysis patients.  

In our study, we described a high frequency of cognitive impairment in hemodialysis patients. At baseline, 75% of the patients suffered from some degree of cognitive impairment, 24% of whom were severe. This is in accordance with previous studies reporting a range from 30 to 80% for cognitive impairment [1, 3, 5, 6, 19].

The most impaired cognitive performance was immediate memory recall, phonemic and semantic fluency, and the best performance was observed in naming. Impaired verbal memory tests involving dialysis patients have been previously reported [8, 20–22]. Other studies found significant differences in cognitive impairment in executive functions, processing speed, word fluency and short-term verbal and non-verbal memory capacity [23, 24]. One study also showed that all patients performed worse in all cognitive domains, especially in memory recall and executive functions [25]. Most of these studies evaluated the decrease in cognitive performance using the MMSE [26], a short neuropsychological screening test that is able to detect progression of cognitive impairment in hemodialysis patients. As the MMSE may underestimate the extent of cognitive impairment, we used the more comprehensive CERAD battery.

Patients with ESRD displayed a higher prevalence of vascular risk factors, such as hypertension and previous stroke, than the general population without CKD [27]. Vascular disease is a more likely cause of cognitive impairment than Alzheimer’s disease in hemodialysis patients [28]. Surprisingly, the study population (with and without cognitive deficit) did not differ significantly with regard to the common vascular risk factors. In the literature it is reported that there is a significant difference in the prevalence of hypertension in patients with cognitive impairment [29], however this was found in a pre-dialysis CKD population. Furthermore, the history of stroke or TIA did not differ significantly in the various groups of our patients with different degrees of cognitive impairment. One reason for this could be that dialysis patients tend to have silent cerebral infarctions [30]. However, as not all patients had MRI imaging available, we could not prove this hypothesis.

Overall, our results suggest that an underlying vascular disease is not the main determinant of cognitive deficits, and also suggest to consider further elements for explaining the cognitive decline in hemodialysis patients.

Moreover, anemia, secondary hyperparathyroidism, dialysis disequilibrium and uremic toxins have also been reported as causes of cognitive impairment in CKD [31], as well as dialysis duration [32]. Dialysis vintage (months) and duration of the dialysis session (hours) showed no association with cognitive performance in our patients. Taking into account that all patients showed good dialysis efficiency, as measured by a Kt/v > 1.2, a negative impact of lower dialysis efficiency on cognitive function cannot be excluded.

We also collected and analyzed some relevant laboratory data. One study showed that dialysis patients with higher hematocrit levels performed better in working memory and attention than patients with lower hematocrit levels [33]. This is in accordance with our results, that showed a positive effect of hemoglobin on the performance in semantic fluency. We also found a positive effect of nicotine on phonemic fluency. There is evidence in literature for an enhancing effect of nicotine on some cognitive functions, like verbal memory and executive functions, nonetheless, nicotine abuse remains harmful in other ways [34].

Lower levels of education are associated with cognitive impairment in our study, as reported by others [32, 35, 36]. This may be explained by the association of lower education levels with poor functional and cognitive reserves.

Interestingly, we have additionally found that the amount of reduction in cognitive performance differed between groups and depended on the initial cognitive level. Decline between CERAD-scores at baseline compared to the scores at one-year follow up was only significant in patients with no impairment at baseline, but not in patients who were already cognitively impaired at baseline. A possible explanation for this could be a floor effect (i.e. not enough variance in already impaired patients).

Depression in hemodialysis patients is very common [37]. To evaluate the frequency of depression and its effect on cognitive performance, the Geriatric depression scale (GDS) was adopted, and in our study depression was found to be significantly associated with cognitive impairment. Other studies have also found similar decline in cognition with the presence of depression [32, 38, 39]. This can be explained by the effects of symptoms of depression on domains of executive functioning and processing speed, in keeping with previous studies [39].

Of note, depression is often under-diagnosed in patients with CKD [40]. Only 3.9% of the unimpaired patients suffered from depression, whereas 9.8% (mild cognitive impairment) and 10% (severe cognitive impairment) of our patients fulfilled the criteria for depression. Estimates for the prevalence of depression in patients with CKD and ESRD range widely from 20 to 40% [37, 40]. In contrast, only a low percentage of our patients were considered as suffering from depression. A possible explanation could be the different methods for the diagnosis of depression in the studies. In our study, we used the GDS, which is a short and inexpensive instrument for measuring depressive symptoms in older adults. However, the GDS can only be used as a screening instrument. The application of more comprehensive depression scales like the Beck Depression Inventory or the Montgomery Asberg Depression rating scale may lead to different conclusions.

Some studies reported a time testing effect with optimal function 24 h after dialysis and worsening with time since the last dialysis session [21, 41–44]. The first studies were conducted when acetate dialysate was still in use. A recent study was able to rule out a significant time-dependent effect, without differences when the patients were tested during or after dialysis [45].

Cognitive performance in hemodialysis patients depends also on the testing environment, and the performance was better when tests were carried out in a separate room [46]. In our study we used the real dialysis setting (a room with two or three other patients).

Our study carries some limitations. The test environment may not have been optimal, but having a quiet place for each patient during the testing was not possible. On the other hand, test conditions reflected the usual dialysis setting. Discussions with patients, changes in medication and important decisions are made during dialysis.

Monitoring the change in cognitive performance after 1 year of dialysis is frequently employed (Ref. [47]), however, it would be interesting to test the changes over a longer period of time. Finally, it has to be mentioned that the CERAD test battery is widely used to detect cognitive impairment, but has not been specifically validated in dialysis patients.

In conclusion, creating a neurocognitive profile in hemodialysis patients is important as the prevalence of cognitive impairment, which is related to educational level, is high and is affected by depression. As depression has a significant influence on cognitive impairment, its early identification may allow to timely initiate treatment and positively influence cognitive performance. The neurocognitive profile and the definition of the deficits may allow to establish individual training programs to control and reduce cognitive deficits.

## Supplementary Information

Below is the link to the electronic supplementary material.

Supplementary file1 (DOCX 18 kb)

Supplementary file2 (DOCX 16 kb)

## Abbreviations

CERAD: Consortium to establish a registry for Alzheimer’s disease
CKD: Chronic kidney disease
cp: Constructive praxis
der: Delayed recall
dr: Immediate recall
eGFR: Estimated glomerular filtration rate
ESRD: End stage renal disease
GDS: Geriatric depression scale
MMSE: Mini-mental-state-examination
MoCA: Montreal cognitive assessment
mr: Memory recognition
na: Naming
pf: Phonemic fluency
SBI: Silent brain infarcts
sf: Semantic fluency
TIA: Transient ischemic attack

## Declarations

### Availability of data and material

The datasets used or analyzed during the current study are available from the corresponding author on reasonable request.

### Code availability

No new code was produced in this study.

## References

[1] Murray AM, Tupper DE, Knopman DS, Gilbertson DT, Pederson SL, Li S, Smith GE, Hochhalter AK, Collins AJ, Kane RL (2006). Cognitive impairment in hemodialysis patients is common. Neurology.

[2] Griva K, Stygall J, Hankins M, Davenport A, Harrison M, Newman SP (2010). Cognitive impairment and 7-year mortality in dialysis patients. Am J Kidney Dis.

[3] Fazekas G, Fazekas F, Schmidt R, Kapeller P, Offenbacher H, Krejs GJ (1995). Brain MRI findings and cognitive impairment in patients undergoing chronic hemodialysis treatment. J Neurol Sci.

[4] Kurella Tamura M, Wadley V, Yaffe K, McClure LA, Howard G, Go R, Allman RM, Warnock DG, McClellan W (2008). Kidney function and cognitive impairment in US adults: the reasons for geographic and racial differences in stroke (REGARDS) study. Am J Kidney Dis.

[5] Kurella M, Chertow GM, Fried LF, Cummings SR, Harris T, Simonsick E, Satterfield S, Ayonayon H, Yaffe K (2005). Chronic kidney disease and cognitive impairment in the elderly: the health, aging, and body composition study. J Am Soc Nephrol.

[6] Dasgupta I, Patel M, Mohammed N, Baharani J, Subramanian T, Thomas GN, Tadros G (2018). Cognitive function declines significantly during haemodialysis in a majority of patients: a call for further research. Blood Purif.

[7] Seidel UK, Gronewold J, Volsek M, Todica O, Kribben A, Bruck H, Hermann DM (2014). The prevalence, severity, and association with HbA1c and fibrinogen of cognitive impairment in chronic kidney disease. Kidney Int.

[8] van Zwieten A, Wong G, Ruospo M, Palmer SC, Barulli MR, Iurillo A, Saglimbene V, Natale P, Gargano L, Murgo M, Loy CT, Tortelli R, Craig JC, Johnson DW, Tonelli M, Hegbrant J, Wollheim C, Logroscino G, Strippoli GFM (2018). investigators C-Hs: Prevalence and patterns of cognitive impairment in adult hemodialysis patients: the COGNITIVE-HD study. Nephrol Dial Transplant.

[9] Murray AM, Knopman DS (2010). Cognitive impairment in CKD: no longer an occult burden. Am J Kidney Dis.

[10] Sachdev PS, Brodaty H, Valenzuela MJ, Lorentz L, Looi JC, Wen W, Zagami AS (2004). The neuropsychological profile of vascular cognitive impairment in stroke and TIA patients. Neurology.

[11] Posner HB, Tang MX, Luchsinger J, Lantigua R, Stern Y, Mayeux R (2002). The relationship of hypertension in the elderly to AD, vascular dementia, and cognitive function. Neurology.

[12] Knopman D, Boland LL, Mosley T, Howard G, Liao D, Szklo M, McGovern P, Folsom AR (2001). Atherosclerosis risk in communities study i: cardiovascular risk factors and cognitive decline in middle-aged adults. Neurology.

[13] Arvanitakis Z, Wilson RS, Bienias JL, Evans DA, Bennett DA (2004). Diabetes mellitus and risk of Alzheimer disease and decline in cognitive function. Arch Neurol.

[14] Kurella M, Mapes DL, Port FK, Chertow GM (2006). Correlates and outcomes of dementia among dialysis patients: the dialysis outcomes and practice patterns study. Nephrol Dial Transplant.

[15] Marsh JT, Brown WS, Wolcott D, Carr CR, Harper R, Schweitzer SV, Nissenson AR (1991). rHuEPO treatment improves brain and cognitive function of anemic dialysis patients. Kidney Int.

[16] Petersen RC, Smith GE, Waring SC, Ivnik RJ, Tangalos EG, Kokmen E (1999). Mild cognitive impairment: clinical characterization and outcome. Arch Neurol.

[17] American-Psychiatric-Association: *Diagnostic and Statistical Manual of Mental Disorders*, American Psychiatric Press, New York, 1987

[18] Chandler MJ, Lacritz LH, Hynan LS, Barnard HD, Allen G, Deschner M, Weiner MF, Cullum CM (2005). A total score for the CERAD neuropsychological battery. Neurology.

[19] Findlay MD, Dawson J, Dickie DA, Forbes KP, McGlynn D, Quinn T, Mark PB (2019). Investigating the relationship between cerebral blood flow and cognitive function in hemodialysis patients. J Am Soc Nephrol.

[20] Griva K, Hansraj S, Thompson D, Jayasena D, Davenport A, Harrison M, Newman SP (2004). Neuropsychological performance after kidney transplantation: a comparison between transplant types and in relation to dialysis and normative data. Nephrol Dial Transplant.

[21] Williams MA, Sklar AH, Burright RG, Donovick PJ (2004). Temporal effects of dialysis on cognitive functioning in patients with ESRD. Am J Kidney Dis.

[22] Lux S, Mirzazade S, Kuzmanovic B, Plewan T, Eickhoff SB, Shah NJ, Floege J, Fink GR, Eitner F (2010). Differential activation of memory-relevant brain regions during a dialysis cycle. Kidney Int.

[23] Sanchez-Roman S, Ostrosky-Solis F, Morales-Buenrostro LE, Nogues-Vizcaino MG, Alberu J, McClintock SM (2011). Neurocognitive profile of an adult sample with chronic kidney disease. J Int Neuropsychol Soc.

[24] Yaffe K, Ackerson L, Kurella Tamura M, Le Blanc P, Kusek JW, Sehgal AR, Cohen D, Anderson C, Appel L, Desalvo K, Ojo A, Seliger S, Robinson N, Makos G, Go AS (2010). Chronic renal insufficiency cohort I: Chronic kidney disease and cognitive function in older adults: findings from the chronic renal insufficiency cohort cognitive study. J Am Geriatr Soc.

[25] Tiffin-Richards FE, Costa AS, Holschbach B, Frank RD, Vassiliadou A, Kruger T, Kuckuck K, Gross T, Eitner F, Floege J, Schulz JB, Reetz K (2014). The Montreal Cognitive Assessment (MoCA) - a sensitive screening instrument for detecting cognitive impairment in chronic hemodialysis patients. PLoS ONE.

[26] Folstein MF, Folstein SE, McHugh PR (1975). "Mini-mental state". A practical method for grading the cognitive state of patients for the clinician. J Psychiatr Res.

[27] Collins AJ, Foley RN, Herzog C, Chavers B, Gilbertson D, Ishani A, Kasiske B, Liu J, Mau LW, McBean M, Murray A, St Peter W, Guo H, Gustafson S, Li Q, Li S, Li S, Peng Y, Qiu Y, Roberts T, Skeans M, Snyder J, Solid C, Wang C, Weinhandl E, Zaun D, Arko C, Chen SC, Dalleska F, Daniels F, Dunning S, Ebben J, Frazier E, Hanzlik C, Johnson R, Sheets D, Wang X, Forrest B, Constantini E, Everson S, Eggers P, Agodoa L (2011). US renal data system 2010 annual data report. Am J Kidney Dis.

[28] Bugnicourt JM, Godefroy O, Chillon JM, Choukroun G, Massy ZA (2013). Cognitive disorders and dementia in CKD: the neglected kidney-brain axis. J Am Soc Nephrol.

[29] Wang F, Zhang L, Liu L, Wang H (2010). Level of kidney function correlates with cognitive decline. Am J Nephrol.

[30] Bugnicourt JM, Chillon JM, Godefroy O, Massy ZA: Relationship between silent brain infarction and chronic kidney disease. *Nephrol Dial Transplant,* 24**:** 2005; author reply 2005–2007, 2009. 10.1093/ndt/gfp125 10.1093/ndt/gfp125 19329533

[31] Watanabe K, Watanabe T, Nakayama M (2014). Cerebro-renal interactions: impact of uremic toxins on cognitive function. Neurotoxicology.

[32] Joseph SJ, Bhandari SS, Dutta S. Cognitive Impairment and its Correlates in Chronic Kidney Disease Patients Undergoing Haemodialysis. *J Evol Med Dent Sci,* 8**:** 2818–2822, 2019. 10.14260/jemds/2019/611 10.14260/jemds/2019/611 PMC6800659 31632935

[33] Lee SY, Lee HJ, Kim YK, Kim SH, Kim L, Lee MS, Joe SH, Jung IK, Suh KY, Kim HK (2004). Neurocognitive function and quality of life in relation to hematocrit levels in chronic hemodialysis patients. J Psychosom Res.

[34] Swan GE, Lessov-Schlaggar CN (2007). The effects of tobacco smoke and nicotine on cognition and the brain. Neuropsychol Rev.

[35] Sithinamsuwan P, Niyasom S, Nidhinandana S, Supasyndh O (2005). Dementia and depression in end stage renal disease: comparison between hemodialysis and continuous ambulatory peritoneal dialysis. J Med Assoc Thai.

[36] Gesualdo GD, Duarte JG, Zazzetta MS, Kusumota L, Say KG, Pavarini SCI, Orlandi FS (2017). Cognitive impairment of patients with chronic renal disease on hemodialysis and its relationship with sociodemographic and clinical characteristics. Dement Neuropsychol.

[37] Andrade RCS (2012). Depression in chronic kidney disease and hemodialysis patients. Psychology.

[38] Jung S, Lee YK, Choi SR, Hwang SH, Noh JW (2013). Relationship between cognitive impairment and depression in dialysis patients. Yonsei Med J.

[39] Agganis BT, Weiner DE, Giang LM, Scott T, Tighiouart H, Griffith JL, Sarnak MJ (2010). Depression and cognitive function in maintenance hemodialysis patients. Am J Kidney Dis.

[40] Kimmel PL, Peterson RA (2005). Depression in end-stage renal disease patients treated with hemodialysis: tools, correlates, outcomes, and needs. Semin Dial.

[41] Griva K, Newman SP, Harrison MJ, Hankins M, Davenport A, Hansraj S, Thompson D (2003). Acute neuropsychological changes in hemodialysis and peritoneal dialysis patients. Health Psychol.

[42] Lewis EG, O'Neill WM, Dustman RE, Beck EC (1980). Temporal effects of hemodialysis on measures of neural efficiency. Kidney Int.

[43] English A, Savage RD, Britton PG, Ward MK, Kerr DN (1978). Intellectual impairment in chronic renal failure. Br Med J.

[44] Ratner DP, Adams KM, Levin NW, Rourke BP (1983). Effects of hemodialysis on the cognitive and sensory-motor functioning of the adult chronic hemodialysis patient. J Behav Med.

[45] Karakizlis H, Thiele S, Greene B, Hoyer J (2021). Cognitive performance in dialysis patients - "when is the right time to test?". BMC Nephrol.

[46] Tholen S, Schmaderer C, Kusmenkov E, Chmielewski S, Forstl H, Kehl V, Heemann U, Baumann M, Grimmer T (2014). Variability of cognitive performance during hemodialysis: standardization of cognitive assessment. Dement Geriatr Cogn Disord.

[47] Bossola M, Antocicco M, Di Stasio E, Ciciarelli C, Luciani G, Tazza L, Rosa F, Onder G (2011). Mini mental state examination over time in chronic hemodialysis patients. J Psychosom Res.

