# Supplementary material for: Assessment of cognitive impairment and related risk factors in hemodialysis patients
Source: J Nephrol. 2021 Oct 16;35(3):931–42. doi: 10.1007/s40620-021-01170-3 (PMC8995241; doi:10.1007/s40620-021-01170-3)
Supplement: Supplementary file 1 — Supplementary file1 (DOCX 18 kb) [file 40620_2021_1170_MOESM1_ESM.docx]

Supplement 1: Relationship between test scores and demographic and medical parameters.

|  | **semantic fluency** | **phonematic fluency** | **memory direct recall** | **memory delayed recall** | **memory recognition** | **Naming** | **constructive praxis** | **CERAD Score** | **MMSE Score** |
| --- | --- | --- | --- | --- | --- | --- | --- | --- | --- |
| **Age** | **-.189**** | **-.106*** | **-.362**** | **-.356**** | **-.269**** | **-.286**** | **-.156**** | **-.398**** | **-.249**** |
| **Education** | **.278**** | **.223**** | **.190**** | **.153**** | .089 | **.235**** | **.349**** | **.322**** | **.180**** |
| Systolic blood pressure | -.033 | .045 | .045 | -.006 | .043 | -.020 | .056 | .022 | -.009 |
| Diastolic blood pressure | .061 | .064 | **.126*** | .089 | .074 | .033 | .091 | **.140**** | .040 |
| Pulse pressure | -.047 | .031 | -.045 | -.087 | -.010 | -.025 | -.013 | -.061 | -.021 |
| Time on dialysis, months | -.029 | -.009 | .024 | .042 | .007 | .079 | .012 | .025 | .035 |
| Hours on dialysis per session | .057 | .084 | .044 | .057 | .000 | **.113*** | .045 | .074 | -.037 |
| Equilibrated Kt/v | .001 | -.006 | .007 | -.007 | -.001 | -.007 | -.023 | -.002 | .062 |
| **Haemoglobin** | **.147**** | -.007 | .012 | -.016 | .023 | .034 | .073 | .090 | .001 |
| **Albumin** | .074 | -.054 | .045 | .084 | **.112*** | .083 | .072 | **.114*** | **.122*** |
| Cholesterol | -.039 | -.019 | -.003 | .021 | .097 | -.024 | -.036 | .008 | .000 |
| Calcium-Phosphatprodukt | .047 | .024 | .081 | .097 | .095 | .080 | .002 | .101 | .096 |
| Bicarbonate | -.065 | -.064 | -.050 | **-.113*** | -.049 | -.019 | -.006 | -.081 | -.058 |
| **Sex** | **-.130*** | .001 | .084 | **.120*** | .091 | -.083 | **-.307**** | -.048 | -.032 |
| **Depression** | -.091 | -.043 | -.081 | **-.138**** | -.074 | **-.109*** | -.088 | **-.148**** | -.048 |
| **Arterial hypertension** | -.009 | -.074 | -.090 | **-.135**** | -.028 | **-.142**** | -.041 | **-.100*** | -.013 |
| Coronary heart disease | -.050 | -.036 | -.092 | -.041 | -.086 | .000 | -.014 | -.072 | -.070 |
| Diabetes mellitus | -.059 | -.081 | -.081 | -.070 | -.086 | -.033 | -.032 | -.099 | -.051 |
| **Stroke/TIA** | -.065 | -.059 | -.049 | -.062 | -.040 | -.070 | .000 | -.068 | -.068 |
| Absolute Arrhythmie | .035 | .032 | -.058 | -.030 | -.060 | .064 | -.031 | -.010 | -.024 |
| **Nicotine abuse** | **.163**** | **.169**** | .020 | .056 | -.037 | .089 | **.191**** | **.131**** | .048 |
| Alcohol abuse | .058 | .054 | .003 | .002 | -.003 | .074 | .080 | .046 | -.005 |
| Primary Cause of ESRD | .018 | .059 | .085 | .080 | .056 | -.051 | .053 | .052 | .004 |

*Notes:* Spearmen’s Rho Correlation; **p* < .05; ***p* < .01; ****p* < .001. Kt/v = value of dialysis efficiency; TIA = Transient Ischemic Attack; ESRD = End Stage Renal Dis
