# Supplementary material for: Assessment of cognitive impairment and related risk factors in hemodialysis patients
Source: J Nephrol. 2021 Oct 16;35(3):931–42. doi: 10.1007/s40620-021-01170-3 (PMC8995241; doi:10.1007/s40620-021-01170-3)
Supplement: Supplementary file 2 — Supplementary file2 (DOCX 16 kb) [file 40620_2021_1170_MOESM2_ESM.docx]

**Table Supplement 2:** ANOVA with repeated-measures separately for the differerent degree of impairment on baseline

| impairment one year follow up | dependent variable | F | dfs | Sig. |
| --- | --- | --- | --- | --- |
| non | time | 13.311 | 1,68 | **<.01*** |
|  | Test | 6.182 | 4.825,328.076 | **<.01*** |
|  | Time x Test | 1.276 | 5.074, 345.045 | .273 |
| mild | time | 0.96 | 1,31 | .758 |
|  | Test | 4.056 | 4.685, 145.245 | **<.01*** |
|  | Time x Test | 1.788 | 4.469, 138.544 | .127 |
| moderat | time | 1.341 | 1,83 | .250 |
|  | Test | 11.381 | 3.879, 321.923 | **< .001**** |
|  | Time x Test | .479 | 5.329, 442.267 | .803 |
| severe | time | 1.100 | 1,57 | .299 |
|  | Test | 11.538 | 4.585, 261.368 | **< .001**** |
|  | Time x Test | .815 | 5.159, 294.05 | .543 |

*Notes*: 99% CI: confidence interval;; **p* < .01; ***p* <.001., F= Variance (dispersion squared)
